# Standard hospital blanket warming cabinets can be utilized for complete moist heat SARS-CoV2 inactivation of contaminated N95 masks for re-use

**DOI:** 10.1038/s41598-021-97345-w

**Published:** 2021-09-15

**Authors:** Anand Kumar, Samantha B. Kasloff, Todd Cutts, Anders Leung, Naresh Sharma, Gloria Vazquez-Grande, Tracy Drew, Denis Laframboise, Olivero Orofino, Joe Tanelli, Jay Krishnan

**Affiliations:** 1 Sections of Critical Care Medicine and Infectious Diseases, Departments of Medicine, Medical Microbiology and Pharmacology, University of Manitoba, Winnipeg, Canada; 2 National Microbiology Laboratory, Public Health Agency of Canada, Winnipeg, Canada; 3 Department of Medical Microbiology, University of Manitoba, Winnipeg, Canada; 4 Health Sciences Centre, JJ399, 700 William Ave, Winnipeg, MB R3A-1R9 Canada

**Keywords:** Biological techniques, Biophysical methods, Microbiology techniques, Diseases, Infectious diseases, Viral infection

## Abstract

Shortages of personal protective equipment for use during the SARS-CoV-2 pandemic continue to be an issue among health-care workers globally. Extended and repeated use of N95 filtering facepiece respirators without adequate decontamination is of particular concern. Although several methods to decontaminate and re-use these masks have been proposed, logistic or practical issues limit adoption of these techniques. In this study, we propose and validate the use of the application of moist heat (70 °C with humidity augmented by an open pan of water) applied by commonly available hospital (blanket) warming cabinets to decontaminate N95 masks. This report shows that a variety of N95 masks can be repeatedly decontaminated of SARS-CoV-2 over 6 h moist heat exposure without compromise of their filtering function as assessed by standard fit and sodium chloride aerosol filtration efficiency testing. This approached can easily adapted to provide point-of-care N95 mask decontamination allowing for increased practical utility of mask recycling in the health care setting.

## Introduction

With COVID19 disease activity again reaching new heights throughout the world, extreme shortages of personal protective equipment (PPE), particularly N95 filtering facepiece respirators (FFP) continue to present a substantial obstacle to provision of care^1,2^. We have recently published data showing the relative utility of seven different decontamination techniques to support reuse of N95 respirators beyond their normal single use standard^3^. Several of the assessed decontamination methods are viable in a hospital setting using a centralized processing approach^4^. However, these centralized approaches involving collection of N95 respirators for off-site processing followed by return and re-allocation to end-users frequently do not allow easy return of decontaminated respirators to their original user. Understandably, many health care workers have been reluctant to use a respirator that was previously utilized by another person^5^. Here we report a simple decontamination method using a hospital blanket warming cabinet that could potentially be implemented at the local ward level and would substantially reduce logistic management issues allowing easy return of decontaminated respirators to their previous users.

In our previous paper, we demonstrated moist heat application of 75 °C for 3 h was sufficient to fully eliminate viable SARS-CoV-2 from N95 respirator material^3^. Moist heat can easily be provided using standard heating cabinets (often used for warming blankets) found on most clinical wards in North American and European hospitals. However, only a minority of these devices are designed to achieve temperatures of 75 °C or more. To our knowledge, virtually all models of generic medical warming cabinets (excluding those designed specifically for warming intravenous fluids and medications) can achieve a temperature of at least 70 °C. We sought to determine whether the application of moist heat at 70 °C could decontaminate six different types of N95 respirators experimentally contaminated with SARS-CoV-2 without degrading fit and filtration efficiency over a series of decontamination cycles.

## Materials and methods

### N95 respirators

Six different respirators were evaluated; because of their scarcity, most of them were obtained from two local hospitals after they had been grommeted for fit testing. They included three molded and three pleated types. Molded types included the 1860, 8210 (3M Company, St. Paul, MN) and 1510 (Moldex, Culver City, CA) models; the pleated included the Aura 1870, Vflex 1804 (3M Company, St. Paul, MN) and Pleats Plus 1054 (Aearo Company, Indianapolis) models.

### Heat treatment of respirators

To create a heating chamber akin to a hospital blanket warmer in a high containment (BSL-3) laboratory, a two shelved, 57 L Model BD 56 standard incubator (BINDER Inc., Bohemia, NY) with its temperature set at 70 °C was used. A small pan (6 in. × 6 in., 2 in. depth) filled with approximately 400 mL water was placed below the bottom shelf the night before the experiment to elevate relative humidity (RH) to the highest passively achievable level. Temperature and RH were recorded using EL-USB-2 Temperature & Humidity Data Logger (Lascar electronics, Erie, PA) which has a measurement range of − 35 to 80 °C, 0–100%RH and an accuracy of ± 0.3 °C, 2.25%RH. For fit testing and integrity testing, whole respirators were exposed to the moist heat by placing them on the shelves above the water pan (external convex surface superior) for a continuous 6 h.

### Quantitative fit testing

Quantitative fit testing was performed in a small room (500 ft^3^) using a PortaCount Pro+ Respirator Fit Tester Model 8038 and FitPro+ Fit Test software (TSI Incorporated, Shoreview, MN). An ultrasonic room humidifier (Honeywell, Charlotte, North Carolina) was used to generate aerosol particles for the testing. The quantitative fit test was performed as per CSA Z94.4-18 protocol^6^, where the ratio of particles inside the respirator to the number of particles outside the respirator was determined to calculate the fit factor by the software. Seven well defined exercises were performed as part of this standardized test: normal breathing, deep breathing, turning head side to side, moving the head up and down, reading a standardized passage aloud, bending up and down, and normal breathing. A respirator that scores a fit factor of minimum 100 for each of the exercises and an average of 100 more was considered a pass^7,8^.

### Filter integrity testing

Metal grommets used for fit testing were sealed with glue on the outer and inner surfaces before testing. Filtration efficiency testing was performed using the NIOSH sodium chloride (NaCl) aerosol method on a TSI 8130A Automated Filter Tester^9,10^. Respirators were fastened to a 3 mm thick aluminum disk using 3M 3792LM hot melt glue, and allowed to fully set for 20 min before being loaded into the TSI 8130A. Respirators were challenged for 5 min at a flow rate of 85 ± 4 L/min with an aerosol of NaCl particles at a concentration not exceeding 200 mg/m^3^.

Due to scarcity during the pandemic, a single respirator of each type was used for both quantitative fit and filter integrity testing. The quantitative fit tester and the filter integrity tester were blinded to the nature of the N95 respirators; i.e. whether moist heat treated or untreated.

### Assessment of SARS-CoV-2 inactivation with dry and moist heat treatments

To determine whether several hours of exposure to dry or moist heat at 70 °C would inactivate SARS-CoV-2, small swatches cut from each of the six respirators were surface contaminated with SARS-CoV-2 virus inoculum. The inoculum was prepared by mixing the virus in a standard tripartite organic soil load (bovine serum albumin, tryptone, and mucin) as per ASTM standard to mimic body fluids^11^. Ten µL of the inoculum estimated to contain approximately 5.0 log TCID_50_ of SARS-CoV-2 was spotted onto the outer surface of each respirator swatch at three different positions. Following 60 min of drying, they were placed (external convex surface superior) on the two shelves of the incubator above the water pan for up to six uninterrupted hours (up to 8 h for the dry heat arm). For moist heat treatments, the pan of water was placed in the incubator the night before the experiments (12 h minimum in advance). Corresponding positive control respirator swatches were concurrently spotted with the same viral inoculum, dried under the biosafety cabinet for an hour, and processed for virus titer determination to account for the effect of drying on virus recovery.

Following heat treatment, virus was eluted from the respirator material by excising the spotted areas on each respirator swatch and transferring each into 1 mL of virus culture medium (DMEM with 2% fetal bovine serum and 1% penicillin–streptomycin). After 10 min of soaking and elution of the material by repeated pipetting, the entire eluate from each excised coupon was transferred to each well of a Vero seeded 6-well plate. Plates were incubated for up to 1 week for signs of cytopathic effects (CPE) of viral growth; final readings were taken by comparing CPE positive wells (cell rounding followed by monolayer detachment) with uninfected control wells (intact monolayer). To confirm absence of viral growth, wells that showed no signs of viral growth (CPE) were sub-passaged by transferring 500 µL of supernatant to a new well of freshly seeded Vero cells.

To determine any potential cytotoxic effect of residues from uncontaminated respirator material on the cell monolayer, negative control respirator swatches were prepared and exposed to various heat treatments without viral inoculum. Eluates from each negative control swatch were collected and plated as described above.

Eluates recovered from positive control coupons were used for viral titer determination in TCID_50_ per Reed and Muench^12^. Additionally, TCID_50_ back-titration of 10 µL of liquid inoculum accompanied each trial to ensure comparable recovery from eluted positive control coupons. The limit of detection of the TCID_50_ assay was 0.8 logs/mL. Results for each treatment indicate mean ± standard deviations of three biological replicates.

### Temperature and RH recorded inside sterilization pouch and paper bag

**Figure 1:**
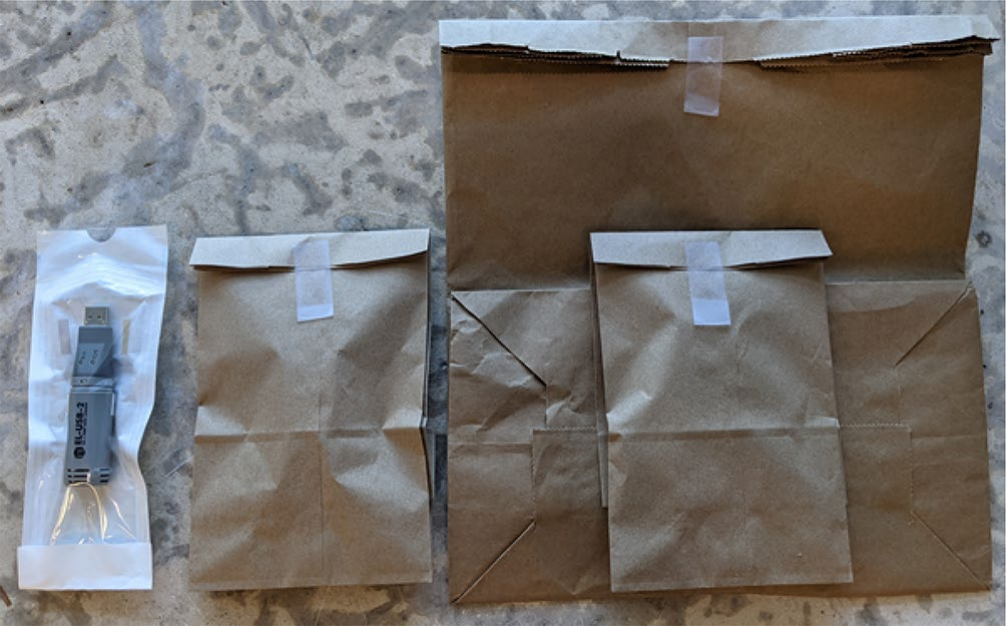
Sealed bags left to right: steam sterilization pouch, single layer paper bag, double layer (grocery and lunch size) paper bags.

In the real-world application, our experience and that of others shows that individual N95 respirators will likely be placed in steam sterilization pouches, paper bags or other containers before being placed in the warming cabinets^13,14^. To mimic this, a temperature/RH logger was placed inside several types of bags in order to ascertain that a N95 respirator in a bag or bags in a warming cabinet would be exposed to the appropriate temperature-humidity profile. The first logger was placed inside a steam sterilization pouch (Chex-All II Instant Sealing Pouch, Propper Manufacturing Company, Long Island City, NY) that was sealed before placing in the warming cabinet. Similarly, another logger was placed inside a single paper grocery-size bag (brown single-layer 140 GSM Kraft paper), its opening folded once and a piece of tape was used to keep the fold in place. In the third configuration, a logger was placed inside two bags; first by bagging in a lunch-size bag (brown single-layer 120 GSM Kraft paper), the opening of which was once folded closed, then placing the smaller bag inside another paper grocery-size bag as before, the opening of which closed in the same manner as before (Fig. 1).

## Results

### Recovery of SARS-CoV-2 from N95 respirator swatches

Back-titration of the 10 µL input virus inoculum resulted in a titer of 4.59 ± 0.1 log TCID_50_. Assessment of positive control respirator surfaces showed nearly complete virus recovery from all six untreated control N95 respirators after drying for an hour, ranging from 4.62 ± 0.1 log TCID_50_ (molded respirators) to 4.44 ± 0.1 log TCID_50_ (pleated respirators). Recovery was not significantly different among the various respirators.

### Viral inactivation by dry or moist heat treatments

**Figure 2:**
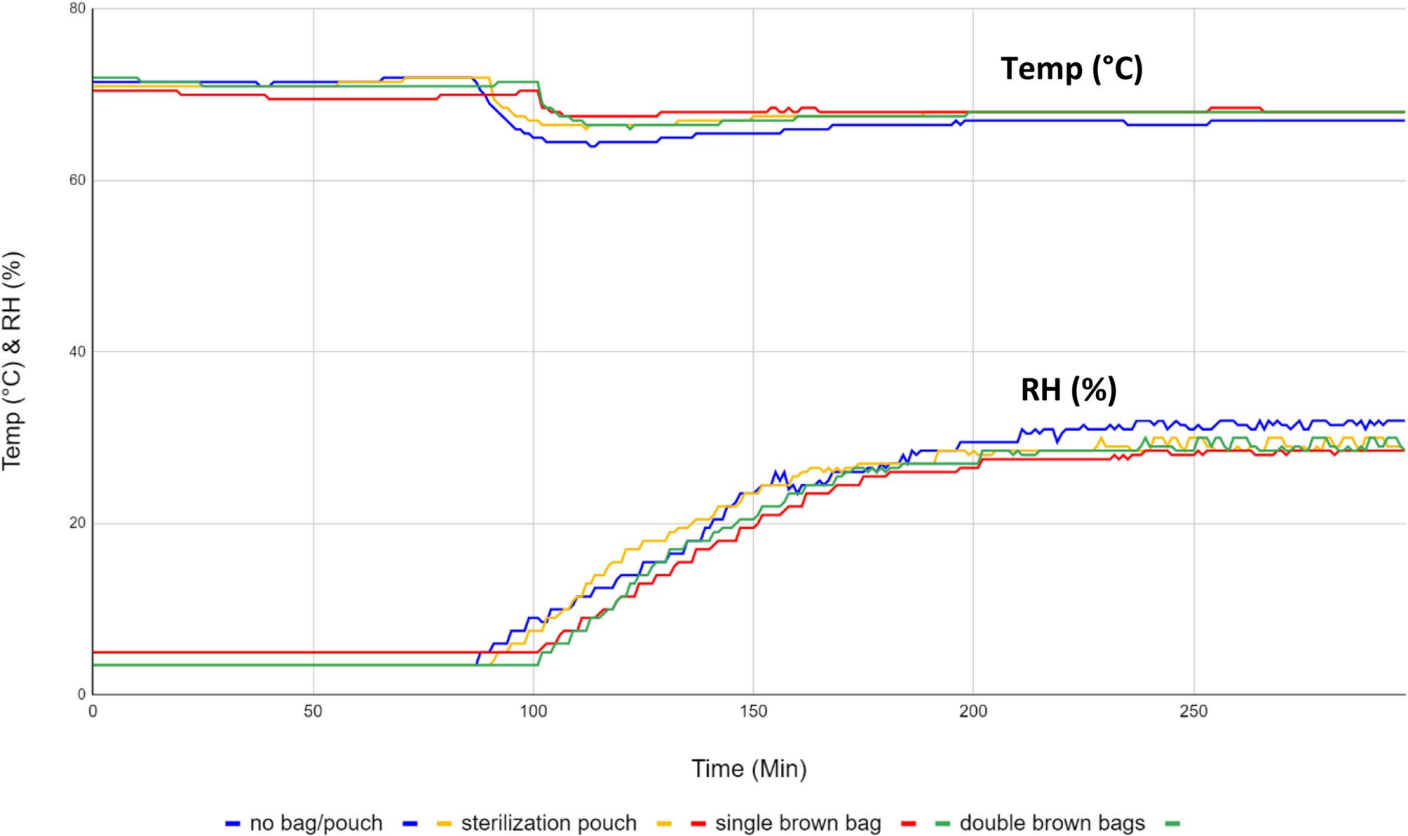
Temperature and relative humidity measured inside the warming cabinet when the temp/RH logger was placed inside a sterilization pouch, inside a single or double bags or no bag/pouch.

Dry heat and relative humidity data measured from the middle shelf of the incubator without the pan of water showed the temperature and RH plateau at 71.5 °C and 3.5% respectively. Initial experimental attempts performed using SARS-CoV-2 contaminated N95 respirator swatches that are exposed to the dry heat failed to inactivate infectious virus, even after 8 h of exposure (Table 1). Placement of a pan containing 400 mL water at the bottom of the incubator increased the RH from it’s drop to < 5% after opening the cabinet to 32% in less than 2 h (Fig. 2); interestingly, at the same time as RH rose, the peak temperature dropped to 67 °C. The temperature and RH remained stable after equilibrating as long as water remained in the pan. Even though 8 h of dry heat treatment only inconsistently inactivated SARS-CoV-2 on the respirator swatches (16 of 18), 6 h of moist heat treatment was sufficient to completely inactivate SARS-COoV-2 virus on all of them (18 of 18) (Table 1). Three hours of moist heat treatment could not inactivate any of the N95 swatches while 4 and 5 h treatments decontaminated 17 of 18 in each group (Table 1). No signs of cytotoxicity was found on any of the eluates collected from uncontaminated negative control respirators.

**Table 1:** Efficacy of heat treatments in decontaminating N95 respirators contaminated with SARS-CoV-2.

N95 respirator	Unexposed^a^	Dry heat		Moist heat			
		6 h	8 h^a^	3 h^a^	4 h	5 h	6 h^a^
3M Aura 1870	+, +, +	+, +, +	+, +, +	+, +, +	−, −, −	+, −, −	−, −, −
Pleats Plus 1054	+, +, +	−, +, +	−, +, +	+, +, +	−, −, −	−, −, −	−, −, −
3M Vflex 1804	+, +, +	−, +, +	−, +, +	+, +, +	−, −, −	−, −, −	−, −, −
3M 1860	+, +, +	+, +, +	+, +, +	+, +, +	−, −, −	−, −, −	−, −, −
Moldex 1510	+, +, +	−, +, +	+, +, +	+, +, +	−, −, −	−, −, −	−, −, −
3M 8210	+, +, +	−, −, +	+, +, +	+, +, +	+, −, −	−, −, −	−, −, −

+ viral growth present, − no viral growth present; each +or − sign represents presence/absence of viral growth from a triplicate set of N95 respirator swatches; viral titer in unexposed control coupons range from 4.62 ± 0.1 to 4.44 ± 0.1 log TCID_50_.

^a^Data from three separate experiments.

### Impact of moist heat on structural and functional integrity

Moist heat treatment did not result in any noticeable change as assessed by visual or tactile inspection. In addition, all six respirators preserved structural and functional integrity of respirators as assessed by PortaCount quantitative fit testing after up to five rounds of moist heat treatments (Tables 2 and 3).

**Table 2:** Average fit factor before and after moist heat treatments.

Average fit factor^a^			
Manufacture, model	Untreated	Moist heat (70 °C × 6 h) 1 treatment	Moist heat (70 °C × 6 h) 5 treatments
3M Aura 1870	186	371	324
Pleats Plus, 1054	226	326	382
3M Vflex 1804	134	135	274
3M 1860	141	106	110
Moldex 1510	224	378	229
3M 8210	205	303	398

^a^A minimum fit factor of 100 is required to pass the test; only one respirator of each type was fit tested.

**Table 3:** Filtration efficiency testing results of N95 respirators after repeat decontamination cycles.

Manufacturer	Untreated		Moist heat 70 °C × 6 h, 5 treatments	
	Pressure drop (Pa)	Filtration efficiency (%)	Pressure drop (Pa)	Filtration efficiency (%)
3M Aura 1870	76	99.7	69	100
Pleats Plus 1054	27	98.6	29	97.7
3M Vflex 1804	48	99.7	39	98.3
3M 1860	82	99.6	76	98.9
Moldex 1510	114	98.8	107	97.8
3M 8210	76	99.4	78	99.5

A single respirator of each type was used for filtration testing following it’s use in fit testing.

## Discussion

In view of the current heavy demand for extended PPE options, methods to decontaminate N95 respirators for re-use is an area of intense review. Although we (and others), have demonstrated several effective approaches to respirator sterilization and re-deployment^3,15^, most methods still have logistic or practical disadvantages that may limit their uptake in the real world. For example the use of UV respirator treatment is limited by the availability of appropriate UV lamps and concern about their ability to deliver sterilization beyond the exposed respirator surface^3^. Autoclave treatment is broadly available but utility is limited to a subset of pleated (rather than molded) construction^3,16^. Vaporized or gaseous hydrogen peroxide methods require relatively expensive and complex devices that may be in limited supply in the current circumstance. Low temperature hydrogen peroxide gas plasma treatment (STERRAD) is effective for at least one standard cycle or two express cycles but damages N95 respirators beyond that^3,17^. Peracetic acid (PAA) fogging may be a viable methodology but is not well known, and aeration of residual PAA is required before mask re-use. In addition, as a decontamination method that has not been commercially developed, there is no application device specifically approved for PAA fogging. Use of the technique requires a customized approach in which the necessary elements are assembled and a standard operating procedure developed. A practical issue with all these methods relates to the logistic impediment of collecting respirators for off-site processing and subsequent re-distribution; this is a barrier though not an insurmountable one given sufficient time, manpower and resources (although these assets may be in short supply during a severe pandemic)^13,18,19^. An additional and substantial problem is that clinical experience and research has shown that N95 respirator users have a strong aversion to re-using respirators utilized by others despite their sterilization (i.e. the “ick” factor)^5^. Therefore, all these sterilization methods are only likely to be successful if the logistics of processing allows for the return of FFPs to their original user^5,19^. A potential solution, as recommended by others, would be a highly localized (ward level) sterilization process where the individual user can be assured that they are re-using the same respirator^5^.

Many viruses, particularly enveloped viruses, are known to be sensitive to the application of moist heat (sub-boiling point heat with an elevated RH). Exposure to temperatures of 55–95 °C for relatively brief periods of minutes to hours can result in inactivation of a large range of human and animal viral pathogens with higher temperatures being associated with more rapid inactivation ^20–23^. Among the human viral pathogens sensitive to heat of < 100 °C are influenza viruses, vaccinia virus, adenoviruses, rotavirus, hepatitis C virus, norovirus and poliovirus among many other human pathogens ^22,24,25^. Similarly, many viruses including influenza are inactivated more effectively with increased ambient humidity^26,27^. Of particular interest, both human and animal coronaviruses are both temperature and humidity sensitive^23,28–30^. They can, as a consequence, be rapidly inactivated by moist heat treatment^20,29^. MERS-CoV causing Middle Eastern Respiratory Syndrome and SARS-Co-V causing Severe Acute Respiratory Syndrome have been shown to be inactivated by temperatures of 56–65 °C for varying durations of 15 min–2 h with increased inactivation with increased humidity ^31–34^. Thermal inactivation of SARS-CoV-2 has also been documented on several different surfaces with increased ambient humidity augmenting thermal viral inactivation ^35–38^.

Our data demonstrate that exposure of SARS-CoV-2-contaminated N95 filtering facepiece respirators to a temperature of 70 °C in the presence of passive humidity for 6 h is highly effective for thermal inactivation of the virus. For a viable, simple, scalable but local solution to the problem of N95 respirator decontamination, it is necessary to consider the aversion of HCWs to re-use of respirators previously utilized by others. While the substantial logistical problem of collecting N95 respirators for offsite decontamination processing is an issue, returning decontaminated respirators to the same end-user may be a near impossible challenge under the current levels of hospital system stress. Fortunately, the decontamination approach described here lends itself to easy adoption in hospitals and other institutions (see “ESM appendix” for operational suggestions). Heating cabinets used for warming blankets are ubiquitous in hospitals and health care institutions throughout the developed world. All the commonly used models are designed to deliver a temperature of at least 70 °C. While they do not typically offer humidity control, we have demonstrated that the placement of a shallow basin filled with water will consistently yield a relative humidity of more than 20%. As we have shown, used N95 respirators (even contained in a paper bag) exposed to the interior of such a cabinet should be effectively decontaminated with 6 h exposure. Using this method, each HCW can manage decontamination and re-use of their own respirator in their local work site within their typical workshift duration of 8–12 h.

Our study suggests SARS-CoV-2 decontamination of respirators requires more time at 70 °C than might be expected based on other studies that did not use any organic soil load^35^. Our results generated using SARS-CoV-2 virus mixed with a standard tripartite soil load are more representative of the clinical environment where virus is mixed with the accumulated oral and respiratory sections potentially deposited over multiple mask uses. The soil load in this study contains an especially high protein content (equivalent to approximately 5% bovine serum^39^) in order to account for the potential accumulation of high protein secretions on N95 respirators after repeat use.

Most other studies that have examined thermal inactivation of SARS-CoV-2 have not been designed to specifically address the question of N95 respirator decontamination and therefore used different surfaces to assess decontamination^40^. However, virus inactivation efficiency is partially dependent on the medium in which or surface on which the virus is suspended or located^40^. Further, in other cases the ambient humidity was either not noted or was not augmented^37^. Although soiling of the contaminated specimen with biological fluids and mimics containing proteins tend to be protective of viruses^23,41^, no studies of N95 respirators utilized soiling as might be expected on used respirators. The recent study by Daeschler et al.^35^, for example, demonstrated that an hour of exposure to 70 °C with 0% relative humidity was sufficient to drive viable virus to undetectable levels on contaminated N95 respirator coupons. However, their coupons were contaminated with SARS-CoV-2 virus without soiling. In a previous study, we demonstrated that exposure of a similar coupon where the inoculum was prepared by mixing the virus in a tripartite soil load (bovine serum albumin, tryptone, and mucin) as per ASTM standard to mimic body fluids^11^ failed to fully inactive the inoculum with 3 h exposure to 70 °C with 22% humidity^3^. Therefore, our results indicating a requirement for 6 h exposure to 70 °C with 32% RH may better indicate the necessary exposure parameters to effect decontamination in the clinical scenario where some degree of soiling with saliva would be expected.

Our study, like any other, has limitations. For example, we did not test pathogens other than SARS-CoV2 that may contaminate masks either acquired from patients (e.g. influenza virus) or carried asymptomatically by the wearer (e.g. *S. aureus* or other respiratory bacteria). Fortunately a wealth of literature demonstrate that most other viral and bacterial pathogens including *S. aureus* and influenza virus are equally or more sensitive to thermal inactivation as SARS-CoV-2^42–45^.

None-the-less, our data should allow for more enthusiastic uptake of a decontamination and re-use approach to increase effective N95 supply. Point-of-care (local ward) level decontamination methods could substantially reduce logistic management issues (collection of N95 respirators for off-site processing followed by re-allocation to end-users) and increase the probability of uptake of a decontamination and re-use approach to increase effective N95 supply.

## Supplementary Information

Supplementary Information.
