# Supplementary material for: Standard hospital blanket warming cabinets can be utilized for complete moist heat SARS-CoV2 inactivation of contaminated N95 masks for re-use
Source: Sci Rep. 2021 Sep 15;11:18316. doi: 10.1038/s41598-021-97345-w (PMC8443586; doi:10.1038/s41598-021-97345-w)
Supplement: Supplementary file 1 — Supplementary Information. [file 41598_2021_97345_MOESM1_ESM.docx]

**Appendix: Operational Tips for Moist Heat Application**

1. Engineering should calibrate the device thermoregulatory function to ensure a minimum target temperature is achieved
2. Ideally, engineering should also check to ensure humidity targets are achieved using a humidity logger
3. If the appliance (hospital blanket warmer/heating cabinet) has excessive external air leaks, sealing sources of air movement may help to achieve targets
4. Hot water should be used for the water pan. This allows achievement of target temperatures/humidity more quickly
5. The ideal volume of water to be used in the pan depends on the quality of the seal of the appliance. If the seal is good with minimal airflow, a few liters will suffice for 24 hours. However, with a poor seal, evaporation loss is a concern and refills will be required. The appliance should be tested to assess the rate of evaporation loss and time to required refill. Ideally, the pan should hold enough water to last somewhat beyond the duration of the sterilization cycle (i.e. 8 hours target)
